# An artificial nociceptor based on a diffusive memristor

**DOI:** 10.1038/s41467-017-02572-3

**Published:** 2018-01-29

**Authors:** Jung Ho Yoon, Zhongrui Wang, Kyung Min Kim, Huaqiang Wu, Vignesh Ravichandran, Qiangfei Xia, Cheol Seong Hwang, J. Joshua Yang

**Affiliations:** 1 Department of Electrical and Computer Engineering, University of Massachusetts, Amherst, MA 01003 USA; 2 Hewlett Packard Labs, Palo Alto, CA 94304 USA; 3 Institue of Microelectronics, Tsinghua University, Beijing, 100084 China; 4 Department of Materials Science and Engineering and Inter-University Semiconductor Research Center, Seoul National University, Seoul, 151-744 Republic of Korea

## Abstract

A nociceptor is a critical and special receptor of a sensory neuron that is able to detect noxious stimulus and provide a rapid warning to the central nervous system to start the motor response in the human body and humanoid robotics. It differs from other common sensory receptors with its key features and functions, including the “no adaptation” and “sensitization” phenomena. In this study, we propose and experimentally demonstrate an artificial nociceptor based on a diffusive memristor with critical dynamics for the first time. Using this artificial nociceptor, we further built an artificial sensory alarm system to experimentally demonstrate the feasibility and simplicity of integrating such novel artificial nociceptor devices in artificial intelligence systems, such as humanoid robots.

## Abstract (web summary)

The development of humanoid robots with artificial intelligence calls for smart solutions for tactile sensing systems that respond to dynamic changes in the environment. Here, Yoon et al. emulate non-adaption and sensitization function of a nociceptor—a sensory neuron—using diffusive oxide-based memristors.

## Introduction

Humanoid robots, especially those equipped with artificial intelligence algorithms, are useful not only for daily life but also for dangerous tasks or space exploration missions where human deployment is unrealistic^1–3^. The sensory system is critical for these robots to collect data from the outside world, to respond to the working environment change and hence to increase the service quality and life time. Nociceptor is a key sensory receptor that recognizes noxious stimuli such as mechanical stress, chemical molecules, extreme temperatures, etc., through which a warning signal (action potential) is generated and delivered to the central nervous system to initiate a motor response that minimizes potential physical damage^4–8^. The nociceptor is only triggered when the amplitude of the electric pulse generated by the external stimulus exceeds a threshold value. A nociceptor does not adapt to the noxious stimuli it has experienced (i.e., no adaptation) while most other sensory receptors (e.g., sight, hearing, taste, smell, touch) do adapt to external stimuli by either gradually or abruptly reducing their sensitivity when they are exposed to a certain external stimulus for a prolonged time. In contrast, the sensitivity of nociceptor increases if the stimulus is excessively intense and results in tissue damage (known as sensitization^9,10^). For an injured tissue, the threshold value decreases and the intensity of response increases, which are called allodynia and hyperalgesia, respectively.

Currently the sensory system in humanoid robots, the so-called tactile sensor (exteroceptive sensors), is built on traditional complementary metal oxide semiconductor (CMOS) devices^11–13^. The tactile sensor is a device that detect the stimulus from the physical interaction with the environment. Although it distinguishes modern robots from predecessors, the end of CMOS scaling is limiting any further improvement especially in size or scalability. In principle, a sensing system consists of a sensor (e.g., thermoelectric or piezoelectric sensors) and a signal processing module, both of which could be an individual nanodevice, leading to high density and low energy operation. While there are more and more nanoscale sensors developed using emerging devices^14,15^, the signal processing module still remains bulky because of its complex functions, which can now be obtained using a single diffusive memristor. Particularly, the key features of a nociceptor such as no-adaptation, relaxation and sensitization have been realized in a single device in this study, which would require multiple complicated CMOS circuit units to achieve if possible at all. Furthermore, CMOS devices also suffer from radiation exposure, which is commonly encountered in the harsh working environment of a humanoid robot.

Here, we report an artificial nociceptor built upon a diffusive memristor. As a two terminal ionic device that uses resistance states to represent information, memristor has been demonstrated for a wide variety of applications ranging from non-volatile memory^16–32^, bio-system emulators^33–36^, analog^37,38,39^ and neuromorphic computing^40–46^, reconfigurable radiofrequency systems^47,48^ to security applications^49–54^. The diffusive memristor^55^, which recently joined the family of memristive devices, is essentially a volatile threshold switching device with switching dynamics that has enabled direct emulation of biological synapse^55^ and neurons, and can be utilized for other applications such as true random number generators^56^ and physical unclonable functionalities as well. The simple device structure with yet very rich switching dynamics can be explored to build an ideal nociceptor. For such a nociceptor, the external stimulus is correspondent with the input voltage applied to the device, and the threshold switching parameters of the device play the role of the threshold function of the nociceptor. The generated output current is linked with action potential, which is induced only by the noxious stimulus and provides the signal to the central nervous system. Intrinsic threshold switching and relaxation properties in a normal condition were used as the typical threshold and relaxation functions of a nociceptor while the tunability of threshold switching properties induced by applying an excessively high electrical stress to the device was linked to the sensitization of the nociceptor. The nature of tunability of threshold switching properties are also discussed with a plausible model based on the device characterization and mechanism study. All important functions of a nociceptor, including the no-adaptation and sensitization, have been demonstrated in a single device at the same time. Furthermore, a simple and yet bio-realistic artificial thermal-nociceptor module was demonstrated experimentally.

## Results

### Working principle of a memristive nociceptor

**Fig. 1:**
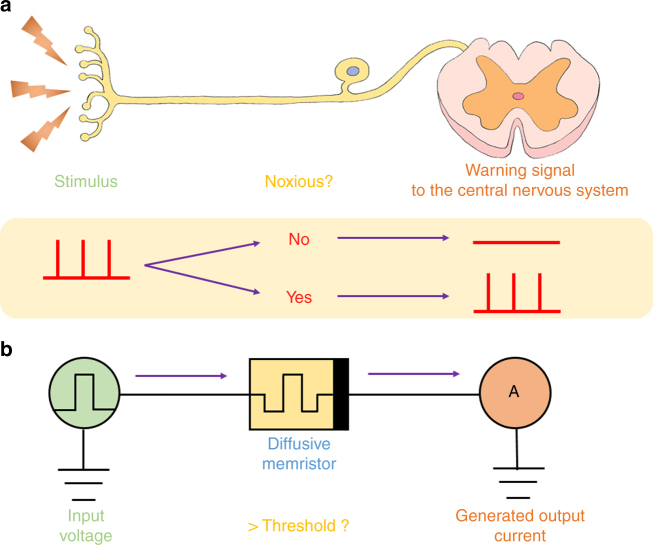
One to one correspondence of the nociceptor system in the human body and an artificial nociceptor circuit consisting of a diffusive memristor (threshold switch). **a** When a noxious stimulus is received from a free nerve ending, the nociceptor compares the amplitude of the signal with a threshold value and decides whether an action potential should be generated and sent to the brain via the spinal cord (central nervous system). **b** An electrical pulse applied on the device emulates the external stimulus. When the pulse amplitude is higher than the threshold voltage of the diffusive memristor, the device is turned ON, and current pulses are generated

Figure 1 illustrates the working principle of our nociceptor and the analogy to its biological counterpart. Nociceptors can be found everywhere in the human body and are located at the end of the sensory neuron’s axon. When a noxious stimulus is received by a neuron located at a free nerve ending, an electrical signal is generated and sent to the nociceptor which compares the amplitude of the signal with its threshold value and decides whether an action potential will be generated and sent to the brain via spinal cord (central nervous system) or not (Fig. 1a). In the memristor nociceptor, an electrical pulse applied on the device emulates the external stimulus. When the pulse amplitude is higher than the threshold voltage of the memristor, the memristor is turned ON (to low resistance state) and a current pulse is detected at the output terminal (Fig. 1b). This process corresponds to the perception of pain in response to a harmful stimulus. The memristor subsequently relaxes back to its original high-resistance state and get ready to respond to the next stimulus. In the bio-system, when the intensity of the noxious stimulus exceeds the threshold of the nociceptor, firing rates of the nociceptor increase with increasing stimulus intensity^57,58^, which indicates how severe the noxious stimuli is. Similarly, our diffusive memristor switches faster (higher frequency) under a higher intensity of the stimulus. In addition, the output current amplitude of our memristor, which could be equivalent to the firing rates of the nociceptor in the bio-system, is proportional to the amplitude of the input electrical pulse. On the other hand, if the input electrical pulse is not strong enough, the memristor stays at its original high resistance state and no current will flow to the output, suggesting the external stimulus is not harmful.

### Diffusive memristor structure and DC electrical behavior

**Fig. 2:**
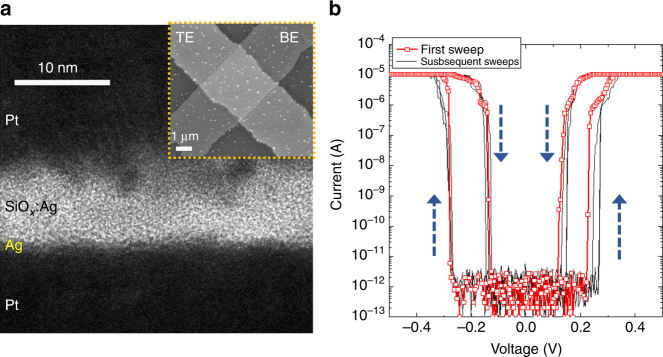
Memristor structure and electrical behavior. **a** High resolution cross-sectional TEM image of a diffusive memristor with a stack of Pt/SiO_*x*_:Ag/Ag/Pt. Inset shows the top-view SEM image of the cross-bar device with a junction area of 2.5 × 2.5 μm^2^. **b** Typical current–voltage (*I*–*V*) curves for the threshold switching under quasi-DC voltage sweeps. The very first switching events are in red and the subsequent curves are in gray. Blue arrows indicate the switching directions

To build the solid-state nociceptor, we used a cross point diffusive memristor device with a junction size of 2.5 × 2.5 μm^2^ and a stack structure of 30 nm Pt/10 nm SiO_*x*_:Ag(11 at. %)/1 nm Ag/15 nm Pt. This device structure is developed from the symmetric diffusive memristor which shows the switching caused depletion of Ag and associated change of threshold conditions (See^55^). A very thin Ag layer (1 nm) was inserted between the Pt bottom electrode and the switching layer as a reservoir of Ag atoms to avoid Ag depletion during switching, which is critical for the “no adaptation” property. Both electrodes were fabricated using photolithography, electron beam evaporation and liftoff, and the switching layer was deposited by co-sputtering of a Ag and a SiO_2_ targets (see Methods for fabrication details). The cross sectional and top-view micrographs of the device are shown in Fig. 2a, from which some small Ag clusters are visible. This phenomenon is probably caused by the out diffusion of Ag clusters in the SiO_*x*_ layer during the deposition process to minimize the total interfacial energy of the material system^55,56^.

Figure 2b shows typical *I*–*V* curves of the bi-directional threshold switching behavior of the Pt/SiO_*x*_:Ag/Ag/Pt device under voltage sweeps (0 to 0.5 V or −0.5 V). A compliance current (*I*_cc_) of 10 μA was adopted during the measurements to avoid damage to the device. As the voltage increased to around 0.25 V (or −0.25 V), the device was turned on and the current rapidly reached the *I*_cc_ level. After removal of the voltage sweep, the device turned itself off spontaneously from its on-state with a sudden current drop at around 0.15 V (or −0.15 V). The response of the device to the quasi-DC voltage sweeps represents the typical threshold switching behavior as previously reported^55,56^. The subsequent switching curves are similar to the first switching curves, indicating that the device is more or less electroforming-free. A nanoscale device, which has a 100 × 100 nm^2^ junction area, shows a similar threshold switching behavior (see supplementary Fig. 1).

### Pulse response of the memristive nociceptor

**Fig. 3:**
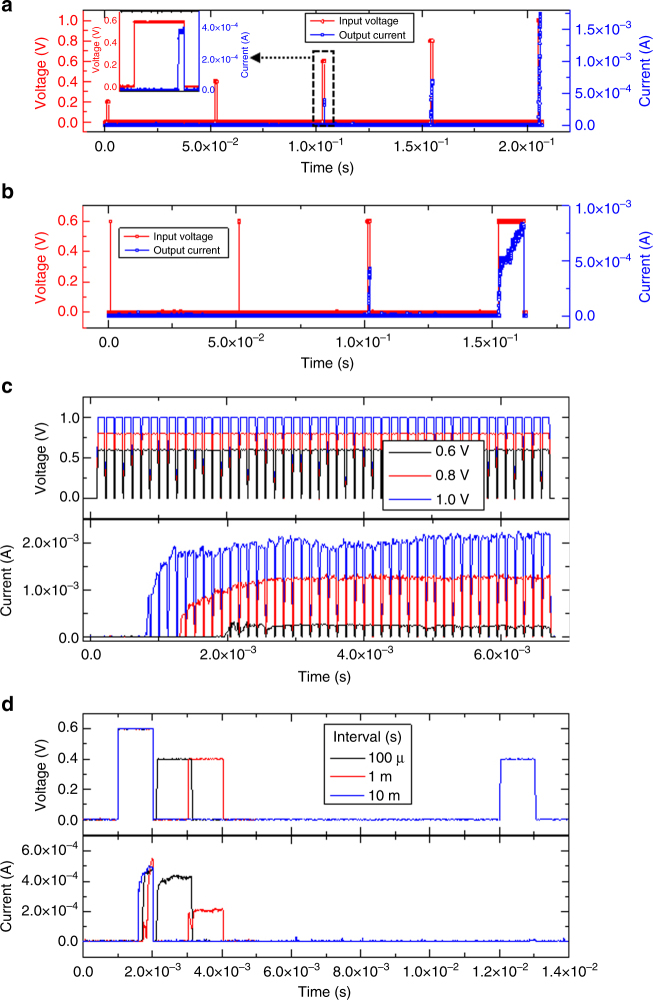
Pulse response of the memristive nociceptor. **a** A train of 1 ms wide input voltage pulses (red curve) of variable pulse amplitudes (0.2 to 1.0 V) and the corresponding output currents (blue curve). A higher input voltage leads to a larger output current. **b** A train of input voltage pulses (red curve) composed of a variable range of pulse widths from 10 μs to 10 ms, with a 0.6 V amplitude, and its output currents (blue curve). **c** Response of the device to multiple number of 100 µs pulses with different amplitudes (0.6, 0.8, and 1.0 V). A higher amplitude results in a shorter incubation time (1.9, 1.3, and 0.8 ms, respectively) in addition to a higher current. The TE was biased while the BE was grounded during all electrical measurements. **d** (upper panel) Relaxation characterization using a 0.6 V pulse followed by a 0.4 V pulse with different interval times between the two pulses  ranging from 100 μs to 10 ms, and (lower panel) the corresponding output currents

The three key features, namely, “threshold”, “no adaptation”, and “relaxation” of a normal nociceptor (undamaged case) were experimentally demonstrated through pulse measurements of the diffusive memristor. The triggering mechanism of a biological nociceptor is highly dependent on the intensity, total time, and the number of stimuli. As such, we used electrical pulses of different amplitudes, pulse widths, and pulse numbers to emulate external stimuli for our memristive nociceptor. With single electrical pulses of 1 ms width, the device was not turned on until the pulse amplitude reached 0.6 V. Further increase of the amplitude to 1.0 V resulted in a larger output current (Fig. 3a; supplementary Fig. 2a–e). This is consistent with the increase of response intensity corresponding to a higher intensity of noxious stimulus in a biological nociceptor. With a fixed pulse amplitude (0.6 V), we observed that a sufficiently long pulse width (1 ms in this case) was necessary to turn on the device, and a longer pulse led to a larger output current (Fig. 3b; supplementary Fig. 2f–i). It is worth pointing out that during these single pulse measurements, a very long interval time (50 ms) was used in between so that the device had enough time to relax back to its resting state.

When a train of electrical pulses were applied to the device, the response is highly dependent on the number of pulses it experienced. Figure 3c shows trains of 100 µs pulses of different amplitudes (0.6–1.0 V, upper panel) and the corresponding output current pulses (lower panel). It is observed that the initial current jump occurred after a certain period of time, which becomes shorter with a higher amplitude of the pulses. This again demonstrates that the threshold property of the device is dependent on the voltage amplitude. Furthermore, it suggests a larger number of pulses are needed to turn the device on if the amplitude is lower. The “threshold” property demonstrated with the memristive nociceptor, which is consistent with that observed in its biological counterpart, can be attributed to the formation of conducting paths (CP; Ag clusters or filaments in this case) within the switching layer. A long enough time and a high enough amplitude are necessary to form such paths to bridge the top and bottom electrodes. Once formed, applying extra number of pulses will sustain the current level without relaxation or degradation (Fig. 3c), indicating that the response intensity does not change or decrease with the repeated input of the same noxious stimuli. This phenomenon is similar to the “no adaptation” characteristic of a nociceptor, and is critical for the human body or a humanoid robot to protect itself against repeated harmful stimuli.

After the noxious stimuli are removed, the nociceptor starts the “relaxation” process. As shown in Fig. 3d, pulse measurements with different interval times (100 μs, 1 ms, and 10 ms) were performed. The device was turned on with a first pulse (0.6 V, 1 ms), and experienced a second pulse (0.4 V, 1 ms) that came after different interval times. The 0.4 V pulse that arrived after the device fully relaxed (10 ms) did not change the device state (blue curves in Fig. 3d). However, if the same stimulation that normally does not turn on the device (e.g., the 0.4 V, 1 ms pulse) was applied before the device fully relaxed, significant current responses were observed (black and red curves in Fig. 3d). This phenomenon resembles the high sensitivity of the nociceptor within the relaxation process, which is also an essential function of the nociceptor. To protect the human body, the nociceptor detects a potentially noxious stimulus with an increased sensitivity right after the nociceptor responds to a pre-noxious stimulus.

### Demonstration of sensitization function in memristive nociceptor

**Fig. 4:**
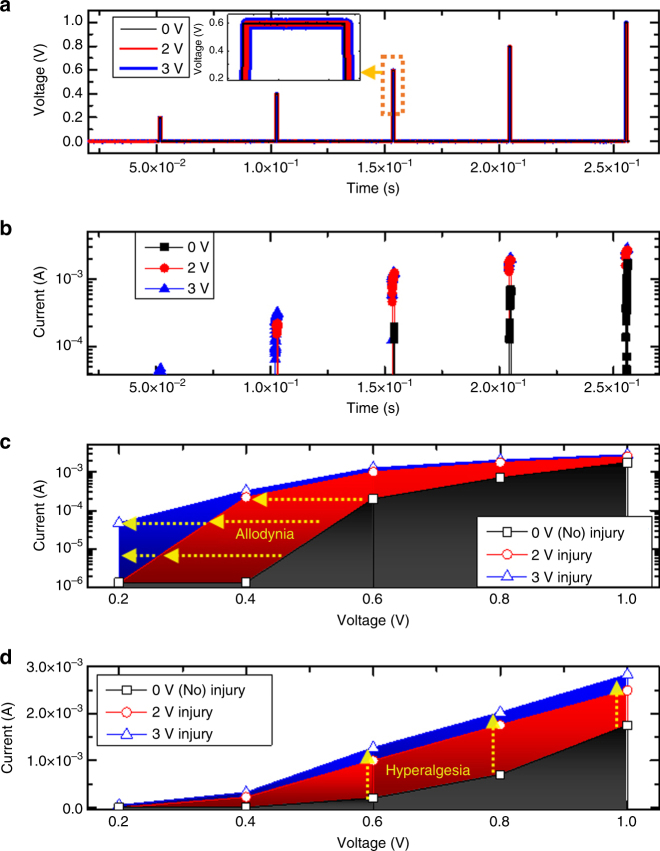
Allodynia and hyperalgesia demonstration of a memristive nociceptor. **a** A train of input voltage pulses composed of a variable range of pulse amplitudes from 0.2 V to 1.0 V, with a 1 ms pulse width applied on a nociceptor subjected to different set pulses (0 V as reference and 2, 3 V with a 1 ms width as injury cases) first and (**b**) the corresponding output currents, respectively. The maximum output currents at a different input voltage amplitudes (**c**) in log scale and (**d**) in linear scale, demonstrating the shift of the ON-switching voltage towards a lower threshold (Allodynia) and the ON current towards higher currents (Hyperalgesia)

As mentioned before, sensitization is a mechanism for a nociceptor to protect an injured area by enhancing the pain sensitivity to noxious stimuli. It can be characterized by “hyperalgesia” and “allodynia”, referring to an increased response to a normally painful stimulus and pain resulting from a normally innocuous stimulus, respectively. To demonstrate the “sensitization” feature of our memristive nociceptor, we first applied pulses with high amplitudes (2 and 3 V, 1 ms width) to the devices, introducing a change that emulates the injury or damage to the nociceptor system. The current response under different input voltages were recorded for devices that have experienced different level of “damages” (Fig. 4a, b). It is clear that the “injured” nociceptors had a higher output current. The maximum output current at different input voltages is plotted in logarithmic and linear scale (Fig. 4c, d). Interestingly, the threshold voltage shifted towards the lower end while the output current shifted higher. This suggests that a smaller threshold voltage is required to turn on a more seriously injured device, reproducing the allodynia and hyperalgesia characteristics in nociceptor. The threshold shift was re-confirmed by the multiple set pulse experiments as shown in supplementary Fig. 3. The required number of pulses, which could be converted into the total pulsing time, in the injury case (after 2, 3 V pulses) to turn on the device is much less than the normal (uninjured) case and the resulting current is much higher, again revealing the threshold change of the device after the high voltage pulse was applied.

To better understand the change of threshold switching properties, electrical characterizations were carefully carried out for normal and “injured” memristive nociceptors. For both cases, the leakage current increased with voltage amplitude (2.0–4.5 V) in the off-state before the ON-switching (supplementary Fig. 4). This result implies that there is either an ionic migration or formation of partial CPs that contributed to the leakage current. This was widely studied in memristor fields and designated as the so-called electroforming process in many material systems including the Ag-mediated switching system^59–63^. In order to verify this assumption in our device, conduction mechanism analysis was performed for several injury-like cases.

**Fig. 5:**
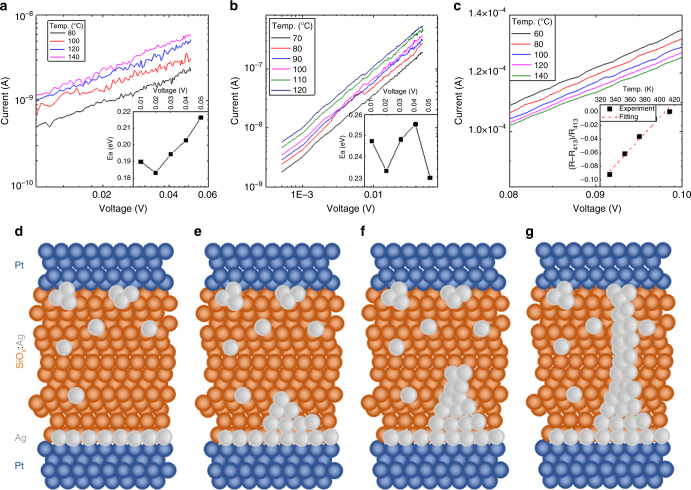
Mechanism study for the change of the threshold properties. Log *I*–Log *V* curves measured at different temperatures of the partially electroformed state obtained by applying (**a**) 2 V (80 ~ 140 °C), (**b**) 3 V (70 ~ 120 °C) pre-pulses and (inset to **a** and **b**). Extracted activation energy for hopping conduction between 0.01 V and 0.05 V ranges. (**c**) Temperature dependent *I*–*V* curves for fully electroformed state obtained by applying 4.5 V pre-pulse and (inset to **c**) (*R*–*R*_413K_)/*R*_413K_ vs. temperature plot. Schematic diagrams illustrating the distribution of Ag ions in (**d**) the normal off state, (**e** and **f**) partially electroformed state (2, 3 V), and (**g**) fully electroformed state (4.5 V)

Figure 5a–c plot temperature dependent IV curves in logarithmic scale for devices that have experienced different forming voltage pulses (1 ms in width, 2.0, 3.0, and 4.5 V in amplitude, respectively). In the partially electroformed cases (2.0 and 3.0 V), the current increases with temperature in the pre-ON-switching state and has a linear relationship with the voltage in the log–log plots as shown in Fig. 5a, b, suggesting a thermally activated electron hopping mechanism. The activation energy for hoping extracted from the temperature dependent data in an Arrhenius plot (ln *I* vs. 1/*T*) is ~0.2 eV (supplementary Fig. 5). However, in the fully electroformed case, an opposite temperature dependence was observed, as shown in Fig. 5c. As the temperature increased, the current value decreased, suggesting the conduction mechanism follows a metallic behavior. This is most likely due to the formation of metallic conducting channels when the high amplitude (4.5 V) pulse was applied. The calculated temperature coefficient is 1.2 × 10^−3^ K^−1^, which was obtained from the (*R*_*T*_–*R*_413_)/*R*_413_ vs. *T* plot shown in the inset to Fig. 5c. This value is close to the reported temperature coefficient of defect-free silver nanowires (2.5 × 10^−3^ K^−1^ ^64^), confirming the metallic nature of the CP. The conduction mechanisms for the normal off-state, the partially and fully electroformed states, are summarized in Fig. 5d–g. In the normal off-state, due to the interface energy minimization effect of Ag in an oxide matrix, Ag clusters are formed at the surface of the SiO_*x*_:Ag layer and a certain amount of silver is distributed in the SiO_*x*_ matrix. The thin Ag layer on top of the Pt BE and the Ag clusters at the interface near the Pt TE could serve as virtual electrodes for the threshold switching on both sides, providing a bi-directional threshold switching for this device. After the high amplitude (but still ≤3.0 V) voltage pulse was applied to the device, the leakage current (current below threshold voltages) increased and the conduction followed a hopping mechanism. This result implies that there would be either residual Ag clusters or conducting filaments, which before fully relaxing reduce the effective gap distance between TE and BE. In addition, these residual filaments (or clusters) can play a role of an electric field concentrator. Both the gap-reduction and the electric-field concentration effects would reduce the switching threshold voltage, resulting in the injury-like case. These partially electroformed states can still fully relax back as time passes, as shown in supplementary Fig. 6, resembling the self-healing process of damaged tissue (nociceptor) in the bio-system. Importantly, this injury-like case (partially electroformed case) is capable of being actively switched back to its normal case by just applying an opposite bias to the device as shown in supplementary Fig. 7. A reset-like behavior (blue curve) was observed in the injured device in the opposite bias direction, recovering the device to its normal off-state. It seems that the injuring process (partial electroforming process) has a directionality and the opposite bias helps to redistribute the condensed Ag atoms, as schematically shown in supplementary Fig. 7. This operation method and its outcome are similar to the “cure” process in actual nociceptors. When the amplitude of an applied voltage is excessively high (≥4.5), an overly strong Ag filament is formed as shown in Fig. 5g. In case this filament cannot be ruptured even by the active approach described above, a permanent damage to the device occurs, resembling the permanent injury of a nociceptor. Both tunability of the threshold switching properties and the active relaxation (reset-like operation) are crucial characteristics of an actively operated nociceptor sensory system.

### Memristive thermal nociceptor

**Fig. 6:**
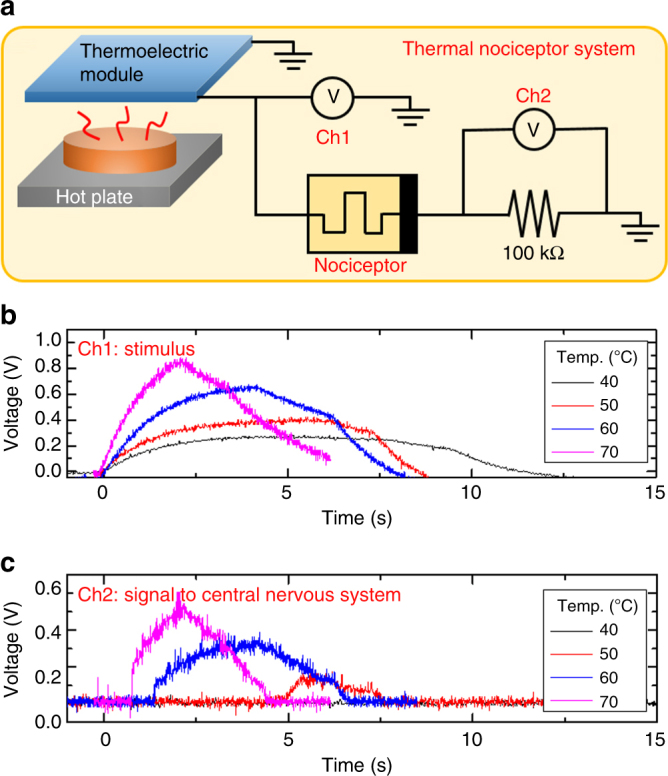
Implementation of a thermal nociceptor. **a** Schematic diagram of the circuit of an artificial thermal nociceptor consisting of a thermoelectric module and the diffusive memristor (threshold switch). **b** The generated voltage from the thermoelectric module and (**c**) the ON-switching and OFF-switching of the threshold switch monitored by Ch1 and Ch2 of the oscilloscope, respectively

With a thermoelectric module connected to a diffusive memristor, we demonstrated an artificial thermal nociceptor. When a thermoelectric plate was heated, a voltage was generated and used as the stimulus signal (Fig. 6a). The maximum amplitude of the generated voltage had a linear relationship with the hotplate temperature (supplementary Fig. 8). If the voltage exceeds the threshold value of the memristor, it will pass a current flow which is sensed by a series resistor. The resistance of the resistor was carefully chosen (100 kΩ in this case) so that the thermoelectric voltage dropped almost completely on the memristor alone before it was turned ON and then dropped evenly on the resistor and the memristor after it was turned ON. The thermoelectric module started to generate a voltage from 0 s (while attaching the module to a hot plate) at each temperature and the voltage started to decrease at a certain time (detaching the module from the hot plate) (Fig. 6b). It was discovered that at 40 °C, the system did not trigger the threshold switch to generate any output alarm signal. While for the temperatures of 50 °C and above, substantial output signals were observed (Fig. 6c), suggesting the onset of an ON-switching event. When the temperature was increased, the amplitude of the signal increased while the onset time of the signal decreased. The threshold voltages observed here are consistent with the observation in Fig. 2b that the threshold voltage of this specific device is about 0.25–0.3 V, which can be engineered by, for instance, changing the Ag concentration (supplementary Fig. 9). Coincidentally, the thermal nociceptors of human body are also triggered against thermal stimuli at around 45 °C.

## Discussion

We proposed and built an artificial nociceptor based on a diffusive memristor with the key functions of a nociceptor implemented all-together in a single device, including “threshold”, “relaxation”, “no adaptation”, “sensitization”, and “cure”. The artificial nociceptor functions based on the unique threshold switching behavior and rich dynamics of the diffusive memristor. After experiencing an injuring condition, the nociceptor has exhibited “hyperalgesia” and “allodynia” as well as passive and active healing processes, again closely resembling an injured bio-nociceptor. We further integrated the device into a thermal nociceptor system that is triggered at a temperature close to the threshold of human nociceptors. The new artificial nociceptor can be readily extended to process other stimuli such as chemical, mechanical, optical, magnetic information. In addition to its promising potentials in normal robots due to its bio-realistic properties and scalability advantages over its CMOS counterpart, our memristor based nociceptor may also be suited for applications in outer space (e.g., next generation Mars Rover) or dangerous environment (e.g., cleanup of Fukushima Daiichi nuclear power plant) because of the proven radhard properties (lacking in CMOS) of memristors^65–67^.

## Methods

### Pt/SiO_*x*_:Ag/Ag/Pt diffusive memristor fabrication

The standard photolithography and lift-off processes were used for top Pt and bottom Ag/Pt electrodes. The 15-nm-thick bottom Pt electrode was e-beam evaporated. The 10-nm-thick SiO_*x*_:Ag(11%)/1-nm-thick Ag layers were deposited by RF sputtering for SiO_*x*_ and DC sputtering for Ag, respectively, on top of the bottom Pt electrode. The 30-nm-thick Pt TE was evaporated after the TE patterning followed by a lift-off process.

### Device characterization

The imaging of the cross-section of the Pt(top)/SiO_*x*_:Ag/Ag/Pt(bottom) sample was carried out using high-resolution transmission electron microscopy (TEM, Titan transmission electron microscopy, FEI). The top-view image of the cross-bar device was obtained by using scanning electron microscope (SEM, JSM-7001F, JEOL). The electrical measurements were conducted using a B1500 semiconductor parameter analyzer (Keysight) for DC measurements and a B1530 (Keysight) module for pulse measurements, respectively. Electrical bias was applied to the TE while the BE was grounded.

### Data availability

The data that support the findings of this study are available from the corresponding author upon request.

## Electronic supplementary material

Supplementary Information
