# Supplementary material for: An artificial nociceptor based on a diffusive memristor
Source: Nat Commun. 2018 Jan 29;9:417. doi: 10.1038/s41467-017-02572-3 (PMC5788850; doi:10.1038/s41467-017-02572-3)
Supplement: Supplementary file 1 — Supplementary Information [file 41467_2017_2572_MOESM1_ESM.pdf]

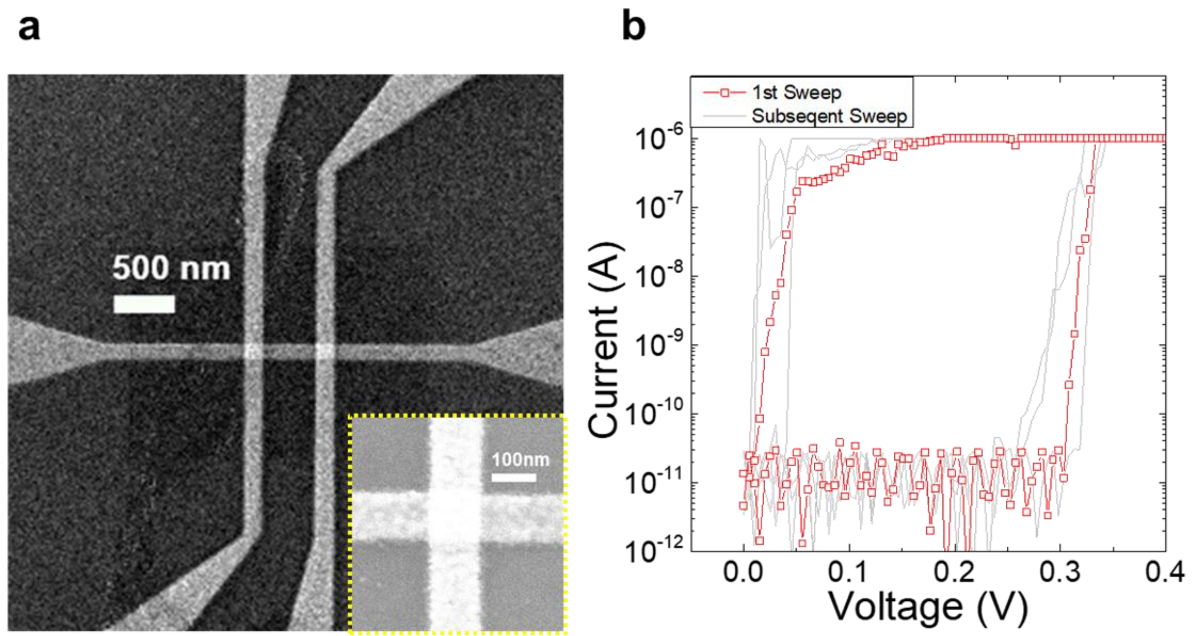

Supplementary Figure 1. **Top-view images and the threshold switching behavior of a nanoscale device.** (a) The top-view and (inset to a) zoomed-in SEM images of a nano-scale device, which has 100 x 100 nm^2 junction area and (b) the threshold switching I – V curves of the nano-scale device, including very first sweep (red curve) and subsequent sweeps (grey curves).

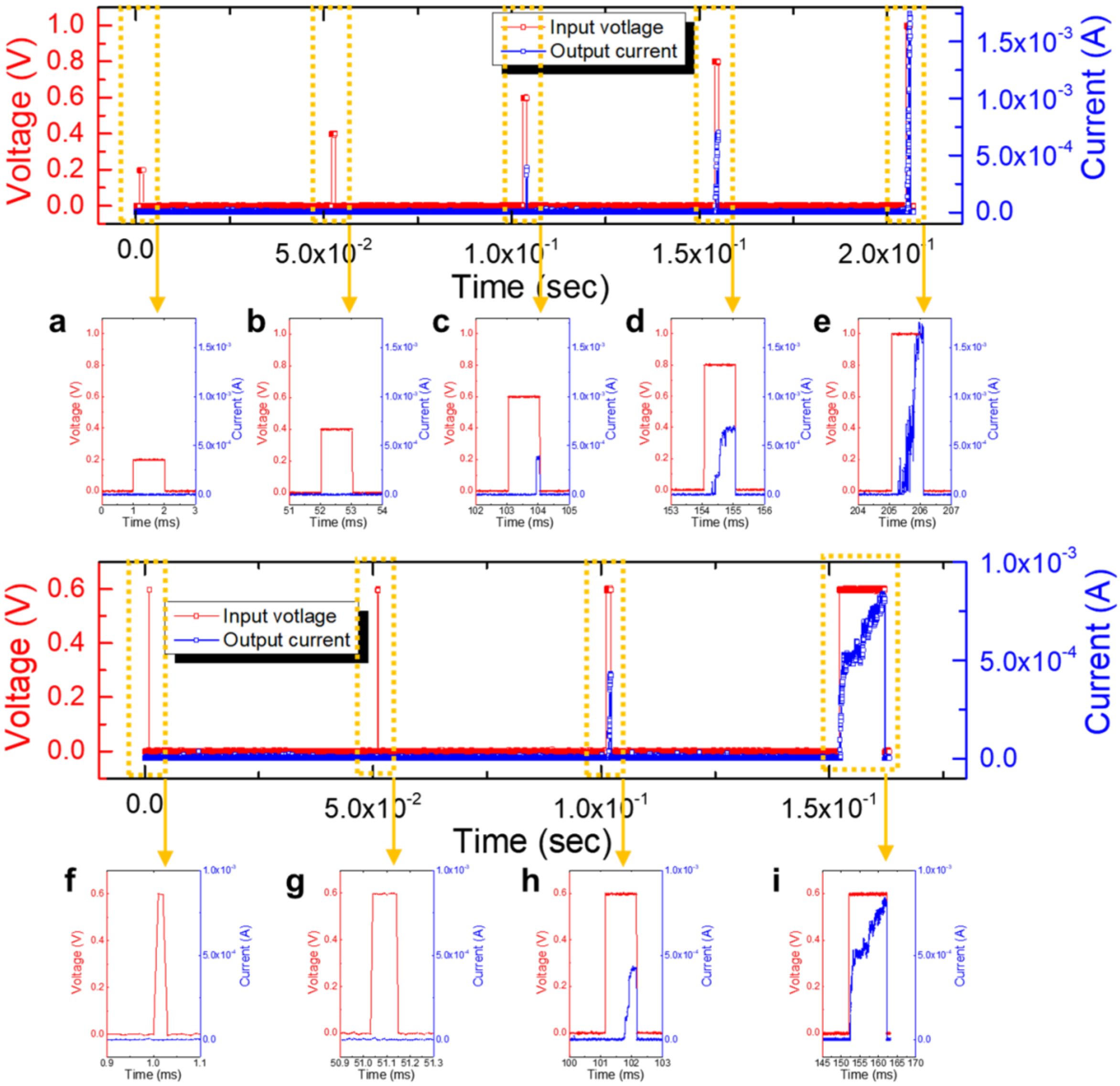

Supplementary Figure 2. **Zoomed-in pulse response of the memristive nociceptor.** (a-e) A train of 1 ms wide input voltage pulses (red curve) of variable pulse amplitudes (0.2 to 1.0 V) and the corresponding output currents (blue curve). A higher input voltage leads to a larger output current. (f-i) A train of input voltage pulses (red curve) composed of a range of pulse widths from 10μs to 10ms, with a 0.6 V amplitude, and its output currents (blue curve).

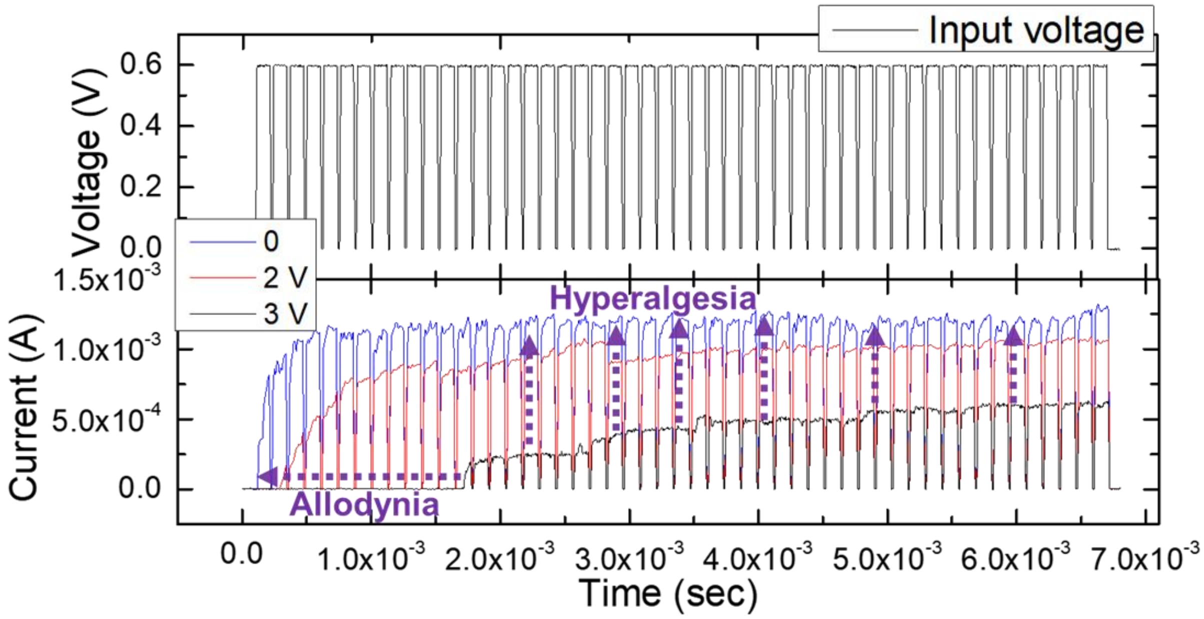

Supplementary Figure 3. **Sensitization functions confirmed by multiple set pulse experiments.** (upper panel) Multiple set pulse experiments with a 0.6 V amplitude and 100μs width in the injury cases (after 2, 3V pulses) and normal (uninjured) case. (lower panel) The output currents for each case, confirming the threshold shift and current increase in the injury cases.

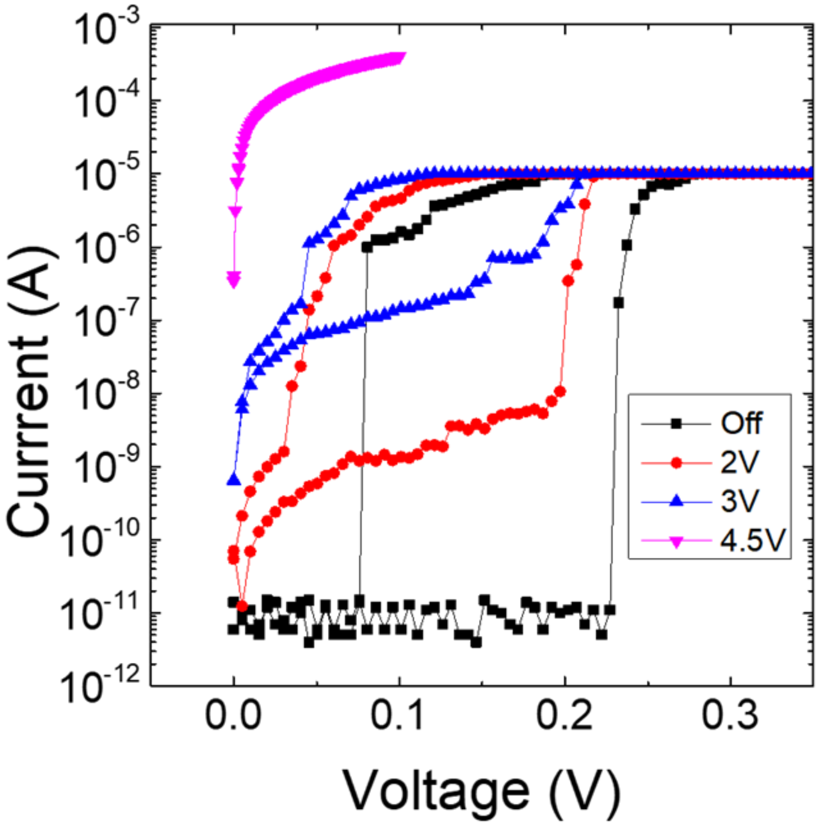

Supplementary Figure 4. **Change of the threshold switching properties after applying the high amplitude pulses (injury cases).** DC threshold switching I – V curves of the device in both normal case and abnormal (injury) cases after undergoing a high amplitude of voltage pulses ranging from 2.0V to 4.5V.

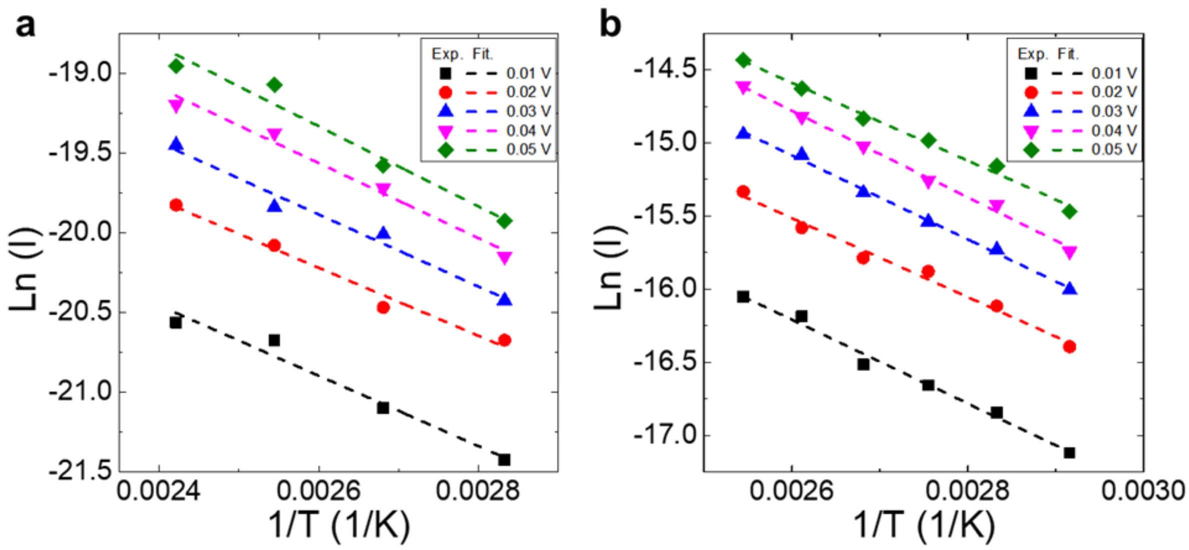

Supplementary Figure 5. **Arrhenius plots for hopping conduction in the partially electroformed states.** Arrhenius (ln I vs. 1/T) plot for electron transport of the partially electroformed state obtained by applying (a) 2V and (b) 3V pulses.

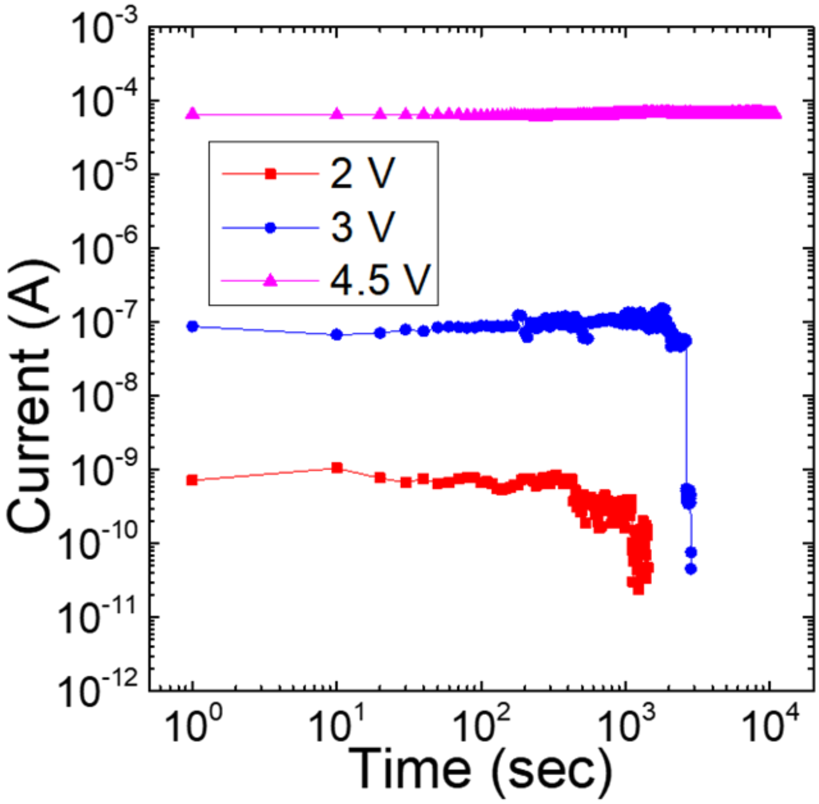

Supplementary Figure 6. **Relaxation properties of the off-currents in the partially and fully electroformed states.** Currents vs. time graph for partially (2, 3V) and fully (4.5V) electroformed states.

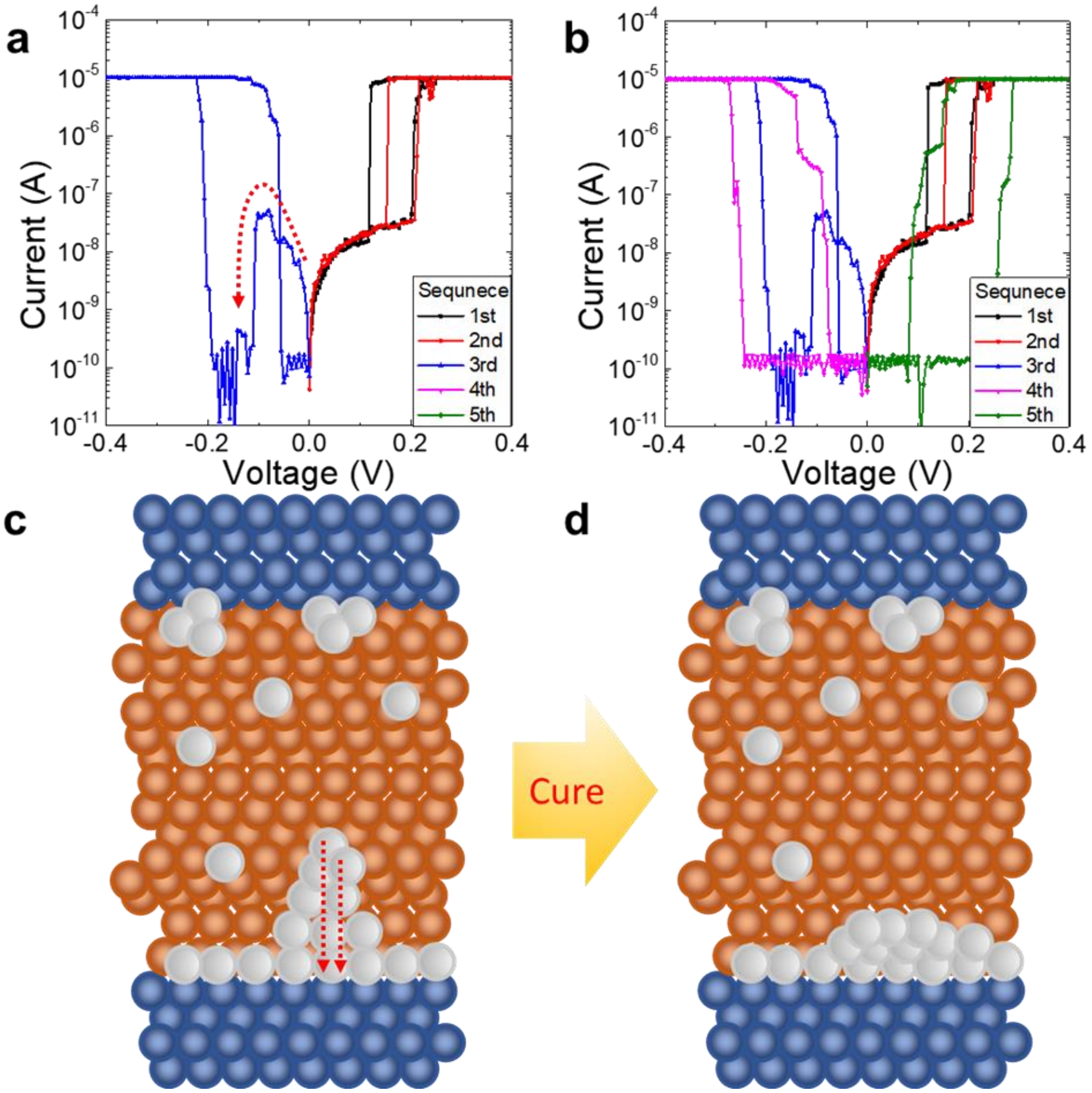

Supplementary Figure 7. DC I – V Operations of active relaxation and its schematic diagrams. (a and b) Sequential DC I – V curves after applying 2V pulse, including the threshold switching curves in partially electroformed state (black and red curves), a reset-like curve (blue curve), and the threshold switching in normal off state after the reset-like behavior in both bias direction (purple and green curves). Schematic diagrams illustrating the (c) partially electroformed state and (d) the normal off state after the reset-like behavior.

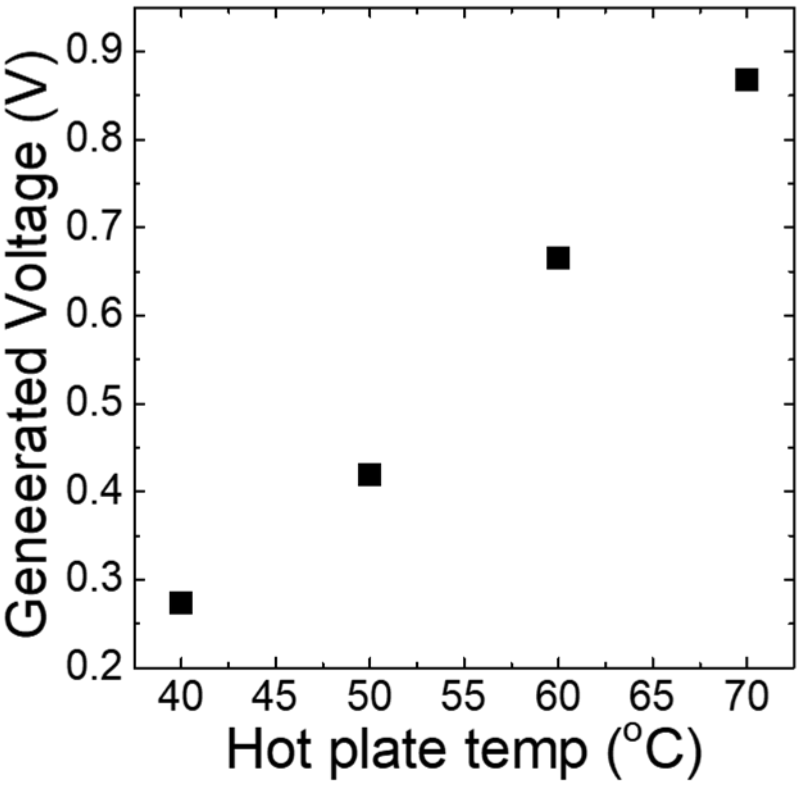

Supplementary Figure 8. The maximum generated voltage as a function of the hot plate temperature.

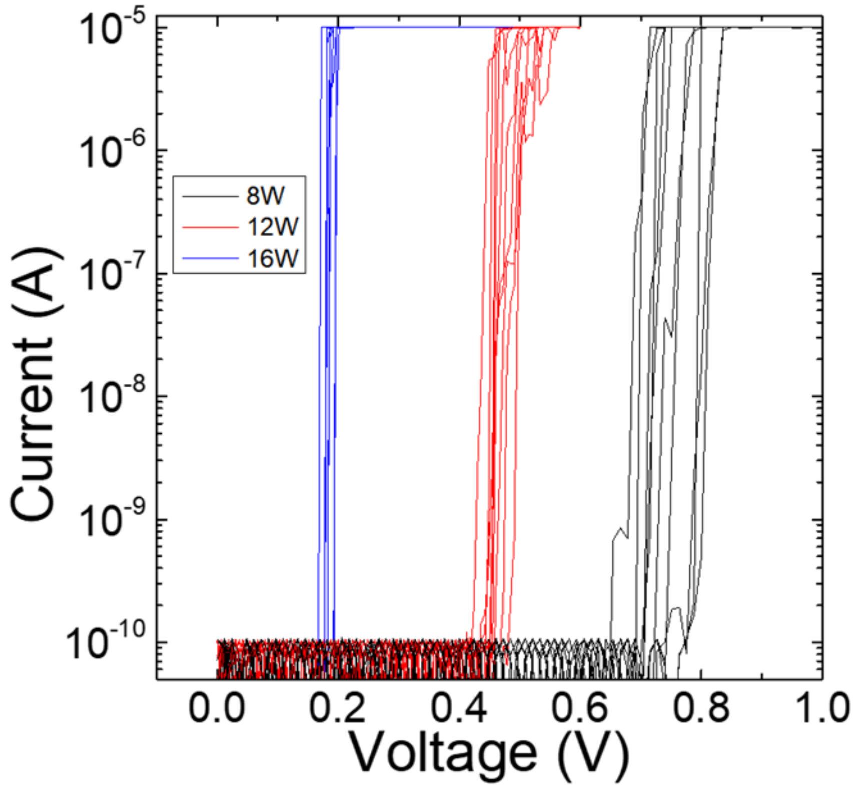

Supplementary Figure 9. **Tunability of the ON-switching properties of the diffusive memristor.** DC ON-switching I – V curves in a SiO_x:Ag-based diffusive memristor with a different sputtering power of Ag ranging from 8W to 16W during the co-sputtering of Ag and SiO_x.
